# Shape Memory Behavior of PET Foams

**DOI:** 10.3390/polym10020115

**Published:** 2018-01-25

**Authors:** Loredana Santo, Denise Bellisario, Fabrizio Quadrini

**Affiliations:** Department of Industrial Engineering, University of Rome “Tor Vergata”, Via del Politecnico 1, 00133 Rome, Italy; denise.bellisario@uniroma2.it (D.B.); fabrizio.quadrini@uniroma2.it (F.Q.)

**Keywords:** shape memory behavior, PET foam, compression mode, shape recovery

## Abstract

Shape memory properties of PET (polyethylene-terephthalate) foams have been evaluated for two different foam densities. Samples were subjected to multiple memory-recovery cycles along three different directions to measure the effect of foam anisotropy on static mechanical and shape memory properties. The memory cycle was performed by uniaxial compression tests at room temperature. Despite these severe conditions, PET foams demonstrated very good shape memory behavior with shape recovery always higher than 90%. Due to cycling, the mechanical performance of foam samples is partially reduced, mainly along the extrusion direction of the foam panels. Despite this loss of static performance, shape memory properties are only partially affected by thermo-mechanical cycles. The maximum reduction is 10% for shape fixity and 3% for shape recovery. The experimental results are particularly interesting considering that compression tests were undertaken at room temperature. Indeed, PET foams seem to be optimal candidates for self-repairing structures.

## 1. Introduction

Shape memory polymers (SMPs) are a very interesting and well-known class of stimuli-responsive materials which have attracted a great deal of attention due to their ability to restore an equilibrium shape upon certain stimulus such as temperature, electricity, pH, ionic strength and light [1]. The non-equilibrium shape can be easily fixed by means of a memorizing step where elastic stresses are frozen into the material structure. Generally, temperature is used to trigger the non-equilibrium shape to the equilibrium shape. In the last decade, SMPs have found a wide range of application in many fields due to the variety of shapes in which they can be manufactured, including sheets, bulks, fibers, and foams. Foam structures improve the shape memory behavior of bulk polymers mainly in terms of compression rate [2,3]. For this reason, SMP foams can be used in the aerospace industry in light actuators [4], and in expandable and self-deployable structures such as wheels for exploration rovers [5], and solar sails [6]. 

At present, numerous methods have been developed to produce SMP foams on the basis of physical (syntactic foams, particulate leaching, phase separation) and chemical principles. Foam structures strongly depend on the production method in terms of cell size, distribution, and extension along the foaming direction. In order to evaluate these differences, the shape anisotropy ratio *R*, defined as the average ratio of cell size in the rise and transverse directions, is considered. Geometrical anisotropy of foam cells leads to anisotropic mechanical properties of foams, with higher strength and stiffness exhibited in the rise direction [7]. The shape anisotropy ratio generally decreases with increasing foam density [8]. In the case of closed-cell rigid PU foams, *R* decreases from 2.5 to 1.7 by increasing the foam density by 1.5 times [9]. By reducing the relative density down to 11–13%, *R* is about 1.6, which is very close to the stiffness anisotropy (1.7), [10]. It would be expected that the anisotropy ratio affects shape memory behavior as well. Polyurethane foams are the first and most studied foams for their shape memory properties [11,12,13], although the effect of the foam structure anisotropy on the shape memory behavior has been never investigated. 

Apart from polyurethane-based foams which are mainly thermosetting, most foams are thermoplastics. However, the shape memory properties related to the foam structure induced by the production process, have not been fully investigated. In particular, the properties of the memory of thermoplastic foams have been evaluated for polycaprolactone (PCL), microbial polyester (poly-hydroxybutyrate-hydroxyvalerate, PHBV), and their mixture (PCL/PHBV) [14]. After training, in the best case, a 100% shape recovery was achieved on 30% compressed PHBV foams. There was a linear dependence of the recovery stress on the temperature, but no correlation with the compression direction or the foam structure was studied. PCL, PHBV, and PCL/PHBV foams are soft at room temperature, and fix the deformed shape by cooling. A different behavior was found in thermoplastic PET (polyethylene-terephthalate) foams, which are rigid at room temperature [15]. PET foams exhibit good shape-memory properties also by performing the memorizing step in cold conditions. This is quite a rare occurrence in the world of SMPs as typically these polymers are strongly damaged when deformed in a rigid state. Small cubic samples were subjected to a memory-recovery cycle which consisted of a cold compression to reduce the foam sample thickness up to 50%, and a subsequent recovery of the shape by heating the samples in a muffle. Final recovery from 97% to 100% was found as a function of the compression direction.

In this study, the recovery properties of PET foams are discussed as a function of the foam orientation. Because of the shape anisotropy induced by the extrusion process of PET foam panels, different behaviors are expected by testing shape memory along the extrusion direction and the two transverse ones. Memory-recovery cycles were performed by means of compression at room temperature (cold compression) and subsequent hot recovery. This thermo-mechanical cycle was repeated three times for each principal direction of the initial foam panel.

## 2. Experimental Methods

Uniaxial compression tests and thermo-mechanical cycles were carried out along three mutually orthogonal directions on PET foams to evaluate the anisotropy of shape recovery and residual stiffness after thermo-mechanical cycling. Samples were extracted from commercial extruded panels, and their properties were measured by a combination of several tests, including differential scanning calorimetry (DSC), dynamic mechanical analysis (DMA), and optical microscopy. Results from these tests were correlated to measured SM behavior under multiple thermo-mechanical cycles. 

### 2.1. Materials

Two commercial PET foam panels (by Point Plastic srl, Colleferro, Italy) were used for experimental tests. The PET foam panels were made of virgin PET resin (Polyclear Refresh PET 1101 by Invista, Charlotte, NC, USA). These types of commercial foam PET panels are commonly used as the core of wind blades and sold by densities. Generally, these PET foams are produced by extrusion with blowing agents (e.g., cyclopentane) and other additives. Blowing agents expand when pressure is released at the extrusion die to form the foam structure. Because of the low cooling rate, high crystallinity is reached. Therefore, PET foams have high structural strength which is combined with low weight and high recyclability. The acquired panels are generally used as core materials for sandwich structures in technical applications such as composite wind blades. 

**Figure 1:**
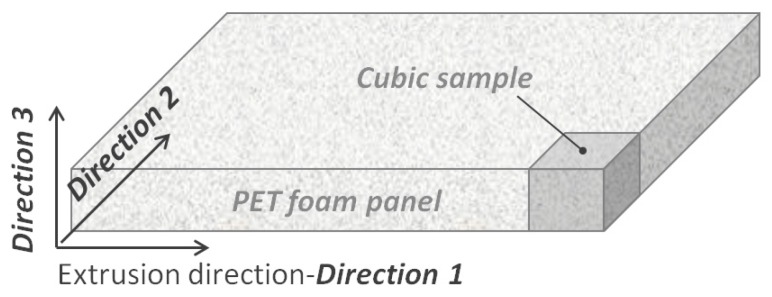
Definition of material directions (and foam orientations) used for PET (polyethylene-terephthalate) foam samples.

Two extruded foam panels (1000 × 600 mm^2^) were used with thicknesses of about 17 and 39 mm, and nominal densities of 65 and 110 kg/m^3^, respectively. In the following, the former foam panel with lower density and thickness is named “low-density foam”, while the latter foam panel is called “high-density foam”. Three material directions were defined on the basis of the panel orientation in the extrusion process as shown in Figure 1. “Direction 1” represents the extrusion direction, whereas “Direction 2” and “Direction 3” are taken in the transverse plane, i.e., the panel cross section. In particular, Direction 2 is aligned with the panel width and Direction 3 with the panel thickness. Cubic samples were extracted from the panels by cutting for mechanical testing and thermo-mechanical cycling. The edge length of the cubic samples was fixed equal to the thickness of the foam panel according to Figure 1, therefore 17 × 17 × 17 mm^3^ samples were cut from the low-density panel and 39 × 39 × 39 mm^3^ samples from the high density panel. 

### 2.2. Foam Morphology

Foam cell morphology was investigated by observing the surfaces of the cubic foam samples by means of a polarized optical microscope (Nikon Eclipse 80i, Nikon Corporation, Minato, Japan). According to the directions of Figure 1, three different planes can be found for observation (1-2, 2-3, 1-3); only the 1-2 plane corresponds to an as-received surface of the foam panel whereas 2-3, 1-3 originated from the panel cutting. In order to assess the shape of the cells, their average size and anisotropy, images were taken in parallel for each direction. Cell dimensions in cross-sectional images were determined in all three main directions. The geometrical ratio was calculated as:

(1) $$R=\frac{1}{n}\overset{n}{\underset{i=1}{\sum }}\frac{h}{b}$$

where *h* and *b* denote cell dimensions in the two directions transversal to each other (e.g., 1 and 2) with respect to the observation plane (1-2), and *n* is the number of cells measured for a given sample. Observations were made on the samples before and after multiple memory-recovery cycles.

### 2.3. Calorimetric and Thermal Mechanical Analysis

Thermal analysis has been carried out on a differential scanning calorimeter (Perkin Elmer DSC6, Waltham, MA, USA) to evaluate the glass transition temperature (*T*_g_) of the PET foams. This value is important to fix the process temperature in the shape recovery step of the thermo-mechanical cycle. According to the ISO 11357:2 standard, *T*_g_ can be calculated as the point of inflection in the step change observed in the heat flow curve. DSC tests were performed in the temperature range between 25 and 300 °C, under nitrogen flux, with a heating rate of 10 °C/min. Calorimetric tests on low-density foams are critical because of their low thermal conductivity and the consequent low sample mass. For this reason, only results for the higher density foam are reported below. In this case, about 3 mg of mass was put into the aluminum crucible. The alternative is pulverizing the foam and using powders for the test but polymer degradation would occur. Nevertheless, the PET polymer formulation was the same for both foam panels and differences were not expected for the *T*_g_. Crystallinity should be different and this datum cannot be extended from the high-density foam to the low-density foam. 

A comparison between the two foams can be made by using dynamic mechanical analysis (DMA). The glass transition temperature *T*_g_. can be also measured by the inflection point of the storage modulus or the maximum peak of the loss factor. Being stiff samples, DMA tests can be carried out easily even if foam collapse under loading can affect results. In this study, the compression mode was used (by Netzsch DMA 242C, Netzsch Instrument Inc., Selb, Germany). Samples with 10 × 10 × 5 mm^3^ approximate size were cut from the panels and tested along the thickness direction (Direction 3). Tests were performed at 5 °C/min from 25 to 170 °C, at the frequency of 1 Hz. 

### 2.4. Thermo-Mechanical Cycling

**Figure 2:**
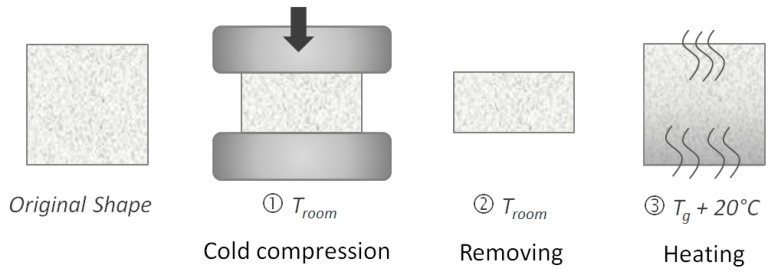
Experimental procedure for shape memory-recovery test.

The shape memory properties of PET foams were evaluated by using thermo-mechanical cycles where the shape was fixed under compression. A universal material testing machine (MTS Insight 5, MTS Systems Corporation, Eden Prairie, MN, USA) was used to make tests along the three directions shown in Figure 1. Figure 2 shows a typical memory-recovery thermo-mechanical cycle. In the first step, a maximum compressive strain ε_m_ was applied at room temperature (25 °C) at 10 mm/min. After load removal, in the second step, the sample was left at room temperature for 1 h in an unconstrained condition so as to stabilize the final strain ε_u_. The difference between the resulting final deformation and the programmed deformation mainly depends on the molecular and shape parameters of the foam [16]. In the third step, samples were heated in the absence of load at the temperature *T*_h_ = *T*_g_ + 20 °C for 80 min in a muffle to recover the strain. A final residual strain (ε_p_) can result. Three samples were tested for each foam and direction, and the thermo-mechanical cycle was repeated three times on each sample. The maximum strain ε_m_ was fixed to 50%. Elastic modulus and maximum compressive stress were extracted from each test. In order to quantify the shape memory properties, shape fixity (*R_f_*) and shape recovery (*R_r_*) were estimated according to Equations (2) and (3), respectively:

(2) $$R_{f}=\frac{\varepsilon_{u}}{\varepsilon_{m}}\times 100$$

(3) $$R_{r}=\frac{\varepsilon_{r}}{\varepsilon_{m}}\times 100$$

where ε_r_ = ε_m_ − ε_p_ is the recovered strain. These parameters refer to the ability to preserve the applied deformation and to recover the initial shape. Shape fixity and shape recovery were evaluated for each thermo-mechanical cycle, direction, and foam so as to evaluate the influence of occurring damages and material anisotropy. 

### 2.5. Conditioning Tests

SMPs may have memory of the processing conditions during manufacturing. PET foams are subjected to strong thermo-mechanical stresses during the extrusion process, and some of these stresses could be released in the first memory-recovery cycle. In order to deepen our understanding of this aspect, some thermal conditioning tests were performed on the low-density foams where higher frozen stresses were expected because of the lower panel thickness. Accordingly, uniaxial compression tests were performed on 17 × 17 × 17 mm^3^ cubic samples after aging in a muffle for 2 h, and compared with results from as-received not-aged samples. Three aging temperatures were chosen (50, 100 and 150 °C). Three foam samples were used for each aging condition. Conditioned and un-conditioned samples were tested at room temperature along Direction 2 of Figure 1, according to the memory step of the thermo-mechanical cycles. 

## 3. Results and Discussion

Understanding the shape memory behavior of foams is a complex task because of the combination of material and structural parameters. Moreover, manufacturing processes strongly influences foam properties, homogeneity and anisotropy. Nevertheless, PET foams have shown very high SM properties also by performing cold memory steps. Typically, SMPs exhibit brittle behavior in the rigid state with very low formability. In PET foams, this peculiar behavior should depend on the combination of high material crystallinity and moderately low glass transition temperature. Crystals always behave like a rigid phase, whereas amorphous PET is partially soft at room temperature and provides structural deformability. On the other hand, molecular mobility is not enough to allow full shape recovery at room temperature. As a result, amorphous PET also allows foam cell collapsing in cold compression but it needs higher temperatures for shape recovery. 

### 3.1. Material Transitions

**Figure 3:**
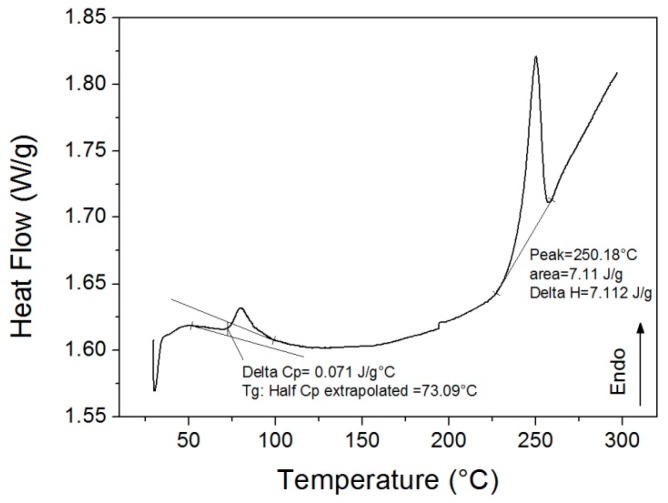
Differential scanning calorimetry (DSC) curve for high-density foam.

By assuming a bulk density of 1.38 g/cm^3^, a relative density of 5% and 8% results for the low and high-density foam. Indeed, most of the foam volume is composed of voids. This is an issue for measuring material transitions with thermal analyses. Figure 3 shows the DSC curve of a high density sample. The *T*_g_ evaluation is affected by the occurrence of relaxation enthalpy but a value close to 73 °C may be inferred. The occurrence of the relaxation enthalpy peak is in agreement with the expected SM behavior as the polymer is able to freeze stresses into the structure. Moreover, that is also evidence of the strong influence of the manufacturing process on the material status. The estimated value of 73 °C for *T*_g_ is in agreement with the expected datum for this kind of resin. A melting peak is also visible in the DSC scan even if its shape and size is probably affected by low sample mass, and foam collapse into the crucible during melting. Nevertheless, a recrystallization peak, before the melting peak, seems to be absent even though it is typical for PET products. It is possible to deduce that the foam itself is close to its maximum crystallinity. After foam extrusion, the panel cooling rate is low because of the very limited thermal diffusivity, and amorphous PET is minimized. 

**Figure 4:**
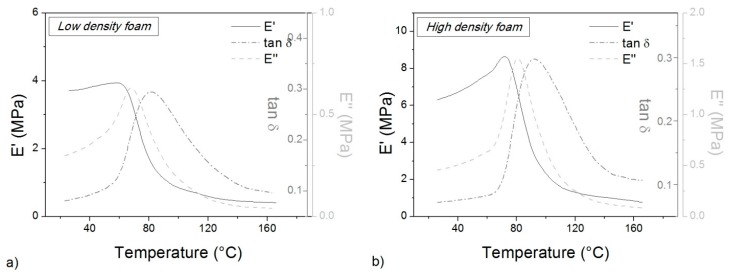
Dynamic mechanical analysis (DMA) curves for low-density (**a**) and high-density (**b**) foams.

DMA tests confirm the data from DSC, as shown in Figure 4. By considering the peak temperature of the loss factor (tan δ) in correspondence with the inflection point of the storage modulus (E’), a *T*_g_ value of 84 °C results for the low-density foam, and 89 °C for the high-density foam. The glass transition is in the same range of the DSC curve but the extracted value is higher because of a small thermal lag. The peak of the loss modulus (E’’) is closer to the DSC value. Unfortunately, results are partially affected by sample collapse under load during heating, as shown by the storage modulus increase before the glass transition. This occurrence could be responsible for the differences in measured *T*_g_ of the low and high-density foam as well as between the DSC and DMA results, even if a perfect superposition is very rare. Nevertheless, the effect of molecular mobility during heating is evident despite the high crystallinity level. The storage modulus of the foam shows a sudden decrease after *T*_g_ even if the melting temperature is far away (250 °C, Figure 3). Also, differences in foam stiffness for the two densities are clear. At 30 °C the storage modulus is 3.7 MPa for the low-density foam, and 6.5 MPa the for high-density foam. However, this difference in the glassy state is completely overcome in the rubbery state (over 150 °C) where foams behave in the same way.

### 3.2. Morphology and Mechanical Properties

**Figure 5:**
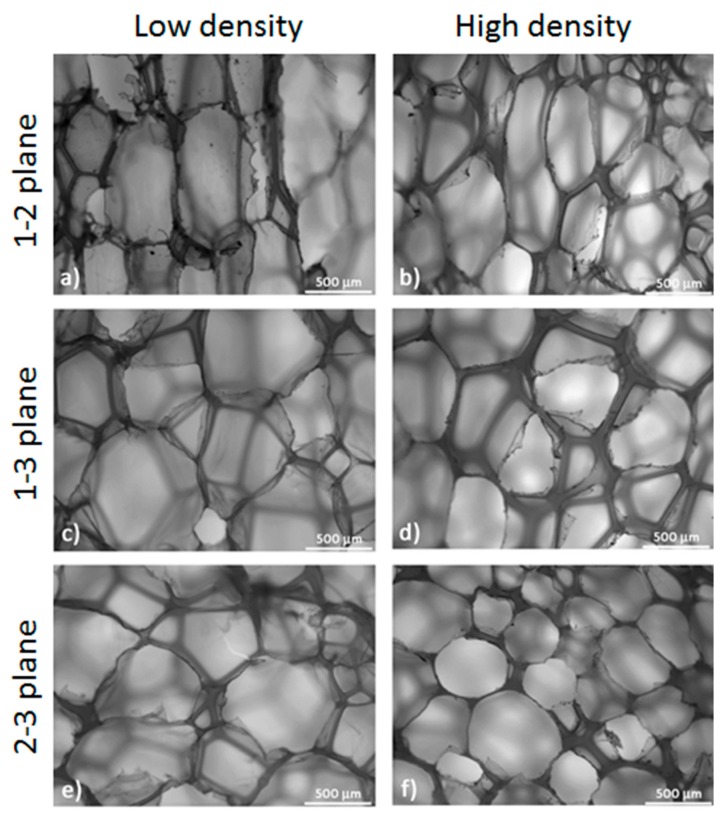
Foam structure for all foam directions and densities.

Micrographs of the two different foams along the three principal directions are shown in Figure 5. The foam structure is closed-cell with an average cell size of 0.54 mm for the high-density foam, and 0.73 mm for the low-density foam. However, the measured cell size is not uniform along all the foam directions. In the 1-2 plane, the average cell size is 0.7 and 0.98 mm, respectively, in the 2-3 plane the average cell size is 0.42 and 0.55 mm, respectively, and in the 1-3 plane, the average cell size is 0.45 and 0.59 mm, respectively. According to these observations, foam cells were mainly elongated along Direction 1 (the extrusion direction) and that is apparent from the comparison between plane 1-2 and 2-3. Cell sizes in plane 2-3 and 1-3 are comparable even if a small increase is present in the latter. A further comparison can be made in terms of the geometrical ratio of the cells along the three principal planes as reported in Table 1. For both foams, the geometrical ratio maximum is in the 1-2 plane, as qualitatively confirmed by Figure 5a,b. Between foams, the cell ratio is higher for the low density sample, where a value of 2.25 is reached. A high geometrical ratio is also present in the 1-3 plane but in this case the maximum value (1.52) is achieved by the high-density foam. However, no anisotropy is observable in the 2-3 plane for both foams. The presence of anisotropy in both the 1-2 and 1-3 planes suggests that cell elongation is not perfectly aligned with the extrusion direction.

**Figure 6:**
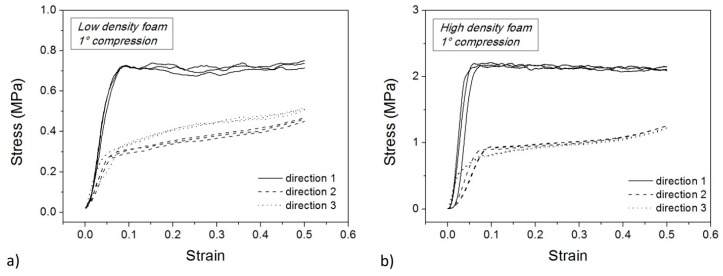
Cold compression tests for low density (**a**) and high density samples (**b**).

Microstructural observations reveal the strong anisotropy of PET foams. It is expected that mechanical and functional performances are affected by this anisotropy. The foam response to external loads depends on the direction of the applied forces, and Figure 6a,b show the compressive stress-strain curves along the three principal directions. These tests correspond to the first loading stage of the memory-recovery cycle. Three samples are shown for each direction. Tests along the same direction are almost perfectly superimposed so as to show the good homogeneity of the initial foam panels.

The effect of the sample orientation is evident. Higher rigidity and higher strength are measured along Direction 1 (the extrusion direction) whereas similar performances are measured in the other two directions (along the panel width and thickness). Along Direction 1, a compressive strength can also be extracted for both foams as the plateau value of the stress. Along the other two directions, a plateau is not visible. Instead, two linear stages are present with different slope. In these cases, a compressive strength can be extracted as the maximum stress (i.e., the stress at 50% strain). Foam densification is not visible along Direction 1 whereas it seems to start after the second linear stage in the other two directions. Higher cell sizes lead to higher material deformability whereas molecular orientation is responsible for stiffness and strength. A difference is appreciable between the width and thickness direction only for the low-density foam because of the high cell size. 

In order to quantify this difference in mechanical properties, the Young’s modulus (E_c_) was estimated from the slope of the initial linear stages, even if non-elastic contributes could be superimposed as well. The maximum stress reached at 50% of the strain was taken as the foam strength (σ_S_). These values are reported in Table 2 in terms of average and dispersion of measured data. The compressive modulus (E_c_) in the extrusion direction is approximately 2 times that in the transverse direction for low density samples. The foam strength in the extrusion direction is approximately twice that of the other two directions as well. Data for the high-density foam are higher but mechanical anisotropy seems to be similar, a part from a lower uniformity in the transverse plane. For example, in the low-density foam, the mean compressive modulus in Direction 3 is 12% higher than in Direction 2. This difference increases up to 42% for the high-density foam. Data for strength are not perfectly aligned due to the occurrence of foam densification during testing. By using the compressive modulus for the evaluation of an anisotropy ratio, a value of 2.1 results along the extrusion direction for the low-density foam, with a result of 2.4 for the high-density foam. These anisotropy ratios are very close to the geometrical ratios of the cells of Table 1. By considering the compressive strength, the anisotropy ratio decreases down to 1.6 and 2.0, respectively. 

### 3.3. Sample Conditioning

**Figure 7:**
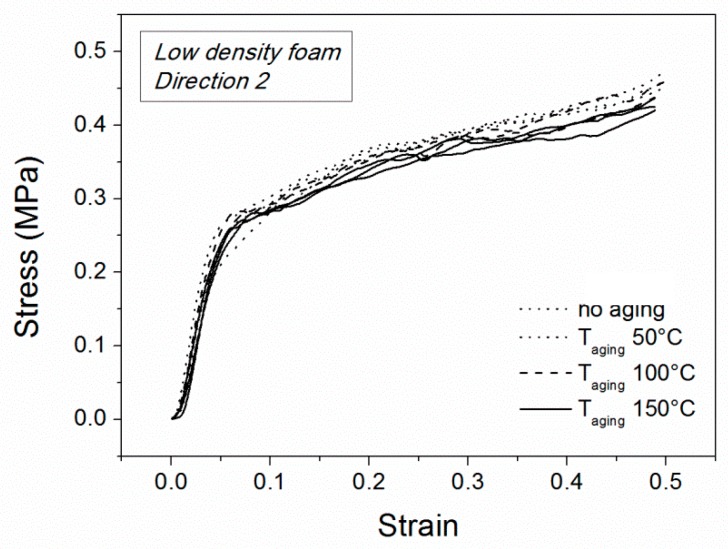
Compression curves after aging for low-density foams along Direction 2.

In order to investigate how the stresses during the extrusion process could affect PET foams in the first thermo-mechanical cycle, some thermal conditioning tests were performed. In particular, three aging temperatures were chosen (50, 100 and 150 °C) and uniaxial compression tests were performed on the low-density foams. The effect of the conditioning treatment on the mechanical performance is shown in Figure 7 for the low-density foam in terms of room-temperature compression curves. The non-aging condition is also reported for comparison. Direction 2 has been chosen for testing because of the low anisotropy ratio. The compression curves are qualitatively similar and they differ only in some quantitative data. 

**Figure 8:**
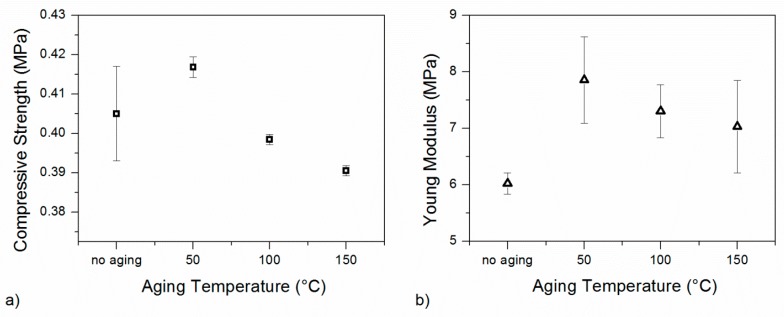
Compressive strength (**a**) and elastic modulus (**b**) for different aging conditions of the low-density foam.

Compression strength and elastic modulus were extracted from these curves and are reported in Figure 8a,b, respectively. By increasing the conditioning temperature, the compression strength decreases but effect is small. Between the maximum (in the absence of aging) and the minimum (aging temperature of 150 °C), an 8% reduction is observed. The same effect seems to disappear in the elastic modulus which is always comparable for all the samples before and after conditioning. Results are comparable with a small material degradation due to thermal aging. This degradation is stronger above the glass transition temperature. Nevertheless, other secondary effects could be superimposed, such as recrystallization, loss of molecular orientation, residual stress recovery and thermal shrinkage. These results suggest that strong material changes during multiple thermo-mechanical cycles should not depend on temperature effects alone but on their combination with applied stresses. 

### 3.4. Shape Memory Properties 

**Figure 9:**
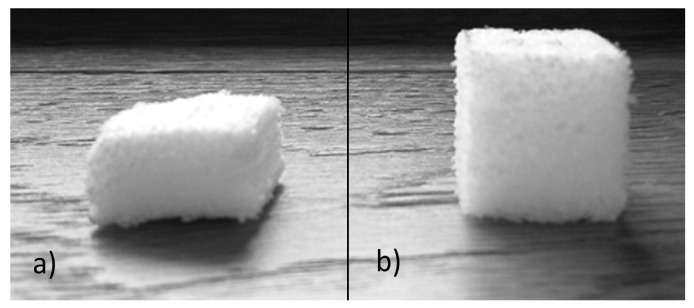
Low density sample after memory (**a**) and recovery (**b**) step of a single thermo-mechanical cycle.

The shape memory properties of PET foams were tested by means of multiple thermo-mechanical cycles. Sample pre-conditioning was omitted because of the negligible effect of the aging conditions. Figure 9 shows a typical foam sample after the memory step which creates a non-equilibrium shape, Figure 9a, and after the recovery step, Figure 9b. The recovery step was performed in a muffle at 100 °C for a total period of 80 min; this time was necessary to complete the recovery phase due to the high porosity of the PET foams. Three samples for each foam and direction were tested in three consecutive memory-recovery cycles for a total of 54 compression tests. Compressive strength and elastic modulus were extracted from each compression curve whereas shape fixity and shape recovery were calculated before each thermo-mechanical cycle according to Equations (2) and (3) respectively. Because of the huge amount of data, it is important to discuss one aspect at a time. 

Table 3 reports shape fixity and shape recovery at the end of the first thermo-mechanical cycle. Shape fixity is always far from 100% because of large strain recovery at the end of the compression step. Material rigidity plays an important role in this mismatch as the highest shape fixity was achieved by the high-density foam which was stiffer. Moreover, for the same foam, higher values were found along the extrusion direction (Direction 1) which was stiffer than both the transverse directions. In fact, the high-density foam in the extrusion direction shows shape fixity of 64%. This value decreases to 52% in the thickness direction (Direction 3), and to 53% in the transverse directions of the low-density foam. Nevertheless, for both foams, low values of shape fixity depend on the elastic strains recovered at the end of the memory step, whereas plastic strains seem to be negligible. This fact is confirmed by the high values of shape recovery, which is always higher than 97% for the low-density foam, and 92% for the high-density foam. Higher shape recovery was achieved in the low-density foam because of the initial lower shape fixity. A small contribution of plastic strain is present, mainly in the extrusion direction of the high-density foam. Being softer, the low-density foam immediately relaxes a part of the applied strain and can also achieve 100% shape recovery along Direction 3. The data in Table 3 confirms that material anisotropy leads to an anisotropic shape memory behavior. Certainly, this anisotropic behavior is more evident for the high-density foam than for the low-density foam for which the shape recovery variability along the three directions is very low. Cell orientation plays a positive role in terms of shape fixity but a negative role in terms of recovery strain. Nevertheless, in comparison with other SMPs, PET foams exhibit excellent properties. In fact, these values for shape fixity and shape recovery were obtained with a cold memory step, while typical testing of other SMPs apply the memory step after heating. 

In applying multiple thermo-mechanical cycles, material and microstructural changes can be expected in the foam because of the combination of heat and stresses. Pre-conditioning tests have shown that material effects only related to thermal aging should be negligible. However, a lot of energy is dissipated into the foam during mechanical loading and this energy can lead to positive or negative effects. 

**Figure 10:**
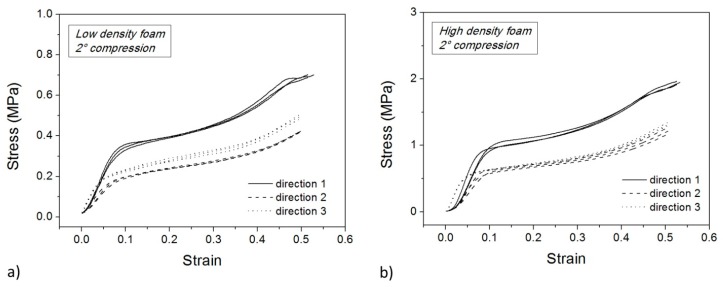
Cold compression tests for low density (**a**) and high density samples (**b**) in the second cycle.

**Figure 11:**
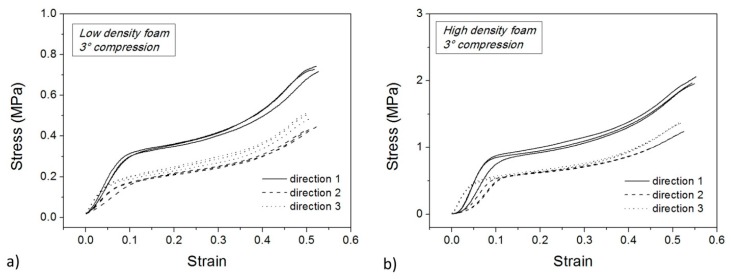
Cold compression tests for low density (**a**) and high density samples (**b**) in the third cycle.

Figure 10 and Figure 11 show the compression step of the second and third thermo-mechanical cycle, respectively, for the low density (a) and high density (b) foam. These curves are similar to those of the first compression test (Figure 6) apart from the occurrence of a more pronounced densification stage at high strain. Also, the stress plateau disappears along Direction 1. In particular, the main differences in compression in Direction 1 are strongly related to the mechanical damage during the first cold compression step. 

**Figure 12:**
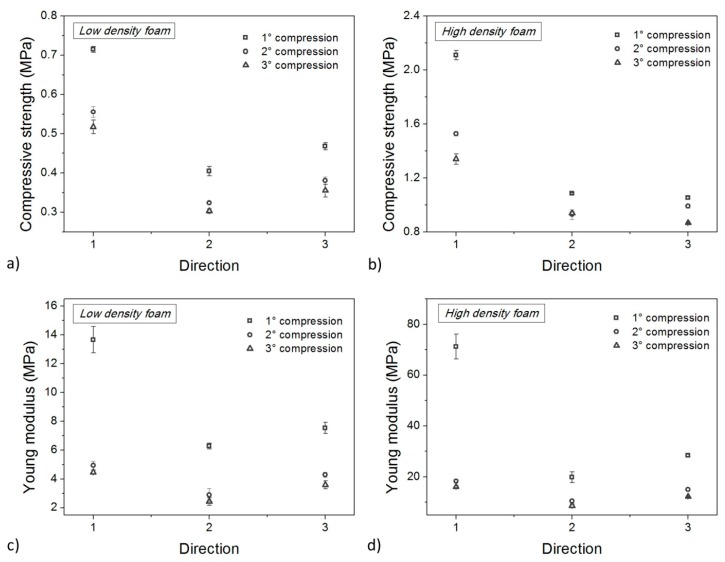
Compressive strength (**a**,**b**) and elastic modulus (**c**,**d**) for low density (**a**,**c**) and high density (**b**,**d**) foam samples in all directions after each thermo-mechanical cycle.

The compressive strength and elastic modulus can also be extracted from the following two compression stages to make a comparison with values from the first stage. Data are reported in Figure 12, which shows that both properties decrease during cycling. For each density, the biggest drop is measured between the first and the second cycle and along the extrusion direction. The loss of properties is also strong in the transverse directions between the first two cycles. Comparing the second and third cycle, a small drop is always present, but not as significant as the previous one. However, in all cases, the effect of panel orientation is stronger than the effect of thermo-mechanical cycling. From this point of view, the first cycle could be seen as a “training cycle” which partially reduces material anisotropies. Nevertheless, mechanical performances at the end of this training cycle are reduced, and continue to minimally diminish in following cycles.

From a quantitative point of view, between the first two cycles, compressive strength along Direction 1 underwent a reduction of about 42% and 48% for low- and high-density foams respectively. Subsequently, between the second and the third cycle, a further reduction of 12% and 14% occurred. Along the same direction, the elastic modulus underwent an even more severe reduction between the first two cycles (64% for low-density foam and 67% for high-density foam) whereas the effect of the third cycle was almost negligible. 

**Figure 13:**
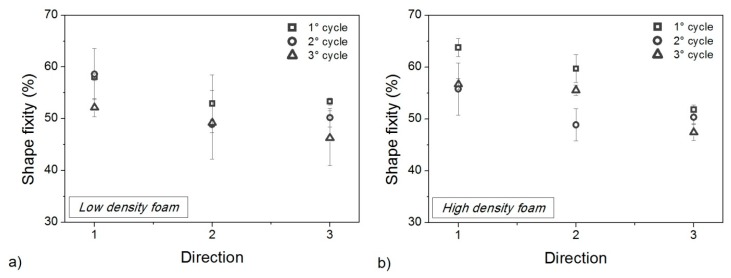
Shape fixity for low density (**a**) and high density (**b**) foam samples in all directions after each thermo-mechanical cycle.

**Figure 14:**
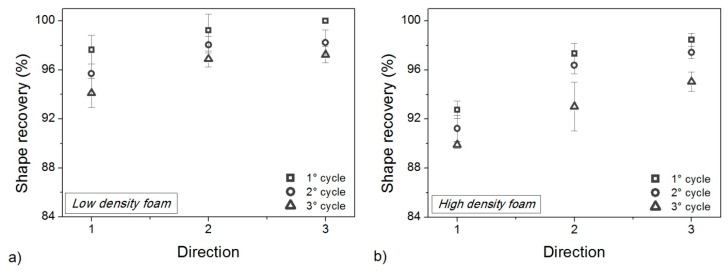
Shape recovery percentage after each memory-recovery cycle for low-density foam (**a**) and high-density foam (**b**).

The effect of foam anisotropy on mechanical performances is very strong and is not erased by thermo-mechanical cycling. It is questionable if a comparable effect is also present for shape memory properties in terms of shape fixity (Figure 13) and shape recovery (Figure 14). According to the static properties (Figure 12), shape fixity and shape recovery are reduced due to thermo-mechanical cycles but the changes are smaller. Dramatic drops, as seen with the compressive strength and elastic modulus, were never observed for *R_f_* and *R_r_*. Foams always preserve good shape memory behavior, and the maximum reduction of *R_f_* after all the cycles, is never higher than 10% (3% for *R_r_*). 

The important role of the panel orientation is confirmed and is never lost due to cycling, although it is not comparable to the static data. Shape fixity in Direction 1 of both foams after the third thermo-mechanical cycle is comparable with Direction 2. Multiple cycles affect mainly material stiffness and strength rather than shape memory properties, and the static properties depend mainly on these characteristics. This can be explained by the fact that static performances depend mainly on the rigid part of the foam material, the PET crystals, which are altered by cyclic loading. In principle, fragmentation of crystallites, alteration of the orientation or a fragmentation of chains in initially pre-oriented asymmetric pore structure (in Direction 1), or the onset/alteration of pore wall buckling/pore wall breaking may be possible contributors to the shifting of the mechanical properties during the first and subsequent cold deformation cycles. Further experiments in this direction may contribute to an improved overall understanding of the observed data. However, shape memory behavior depends on the soft part of the foam material, amorphous PET, which is almost unaltered after thermo-mechanical cycling. Therefore, cell structure and morphology can be affected by cycles but not their ability to store a new shape and to recover the previous one.

**Figure 15:**
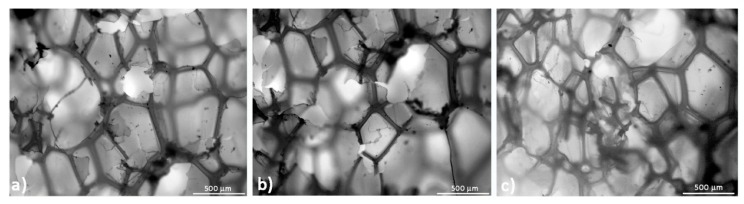
Low-density foam microstructure after each thermo-mechanical cycle: plane 1-2 (**a**); 1-3 (**b**) and 2-3 (**c**).

**Figure 16:**
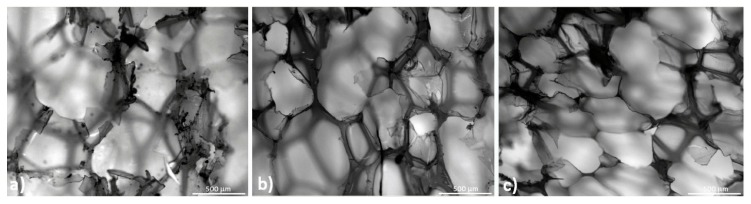
High-density foam microstructure after each thermo-mechanical cycle: plane 1-2 (**a**); 1-3 (**b**) and 2-3 (**c**).

In order to confirm the correlation between foam structure and measured performances, microscopic observations were repeated at the end of each thermo-mechanical cycle. Results are shown in Figure 15 and Figure 16 for low- and high-density foam, respectively. The Figures show that many broken cells and warped cell walls appear during cycling. Because of this damage, it was not possible to estimate the geometrical ratio of cells, as was possible for un-cycled samples. The presence of these ruptures highlights that the sharp reduction of static mechanical properties of low and high density PET foams could be affected by such events. Applied memory-recovery cycles are very severe due to the cold compression step. For this reason, material damage is associated with thermo-mechanical cycles. This damage is not able to erase foam anisotropy, but stiffness and strength are reduced. Nevertheless, despite the cellular materials being partially damaged, their shape memory properties are preserved and sufficient to guarantee a shape recovery of about 90% after three thermo-mechanical cycles. 

## 4. Conclusions

PET foams are not considered shape memory materials but experimental results show that they have the ability to recover their shape after a cold memory step. The combination of shape memory behavior with other already known properties of PET foams, could open a wide variety of applications. PET foams are light, stiff and thermally insulating, and already have a great number of possibilities as a core material in transport, wind turbine construction or shipbuilding. Self-repairing properties could be easily added because of their SM behavior. Moreover, PET foams can be produced from recycled PET and can be 100% recyclable. Nevertheless, the effect of manufacturing processes on foam SM properties needs to be correctly evaluated. For example, the SM behavior of foams is expected to be dependent on the production method and the process conditions. Results also indicate that the SM properties of PET foams are strongly anisotropic. However, after heating, PET foams recovered the initial shape up to 98% in the third thermo-mechanical cycle.

In this study, commercial PET foams were used with two densities (65 and 110 kg/m^3^). These foams were strongly anisotropic with cell orientation along the extrusion direction as underlined by morphological observations. Consequently, the largest elastic modulus and compressive strengths were measured along this extrusion direction. The behavior of the foams in the transverse directions is not exactly isotropic but the related anisotropy is lower. In quantitative terms, the elastic modulus and the compressive strength in the extrusion direction were 2 times higher than that in the two orthogonal directions for the low-density foam, and 3 times higher for the high-density foam. Mechanical anisotropy partially decreases by thermo-mechanical cycling, but the effect of material damage is superimposed. After the first cycle, the mechanical properties are reduced by about 50%. Shape memory properties are less affected by anisotropy and by thermo-mechanical cycling. After three thermo-mechanical cycles, the minimum shape recovery value is about 90% which was measured in the extrusion direction for the high-density foam. This is a good result considering the great number of broken and deformed foam cells after multiple thermo-mechanical cycles. At room temperature, loads or impacts may damage PET foams with a consequent reduction of mechanical properties, but foams preserve excellent shape recovery. Anyhow, a larger number of thermo-mechanical cycles could be useful to better understand the SM properties of PET foams and further studies may investigate this aspect. However, the ability to recover their initial shape after a room temperature deformation extends the possibilities of using such foams. In fact, these properties are suitable for defining a new class of self-repairing materials where shape recovery is associated with self-healing agents. From this perspective, a potential application of SM PET foams could be as the core of self-repairing sandwich structures with composite skins for marine applications, as previously evaluated by the authors [17]. 

## Figures and Tables

**Table 1:** Geometrical ratio of the foam cells for the different planes of observation.

Foam Type	Geometrical Ratio		
	1-2	1-3	2-3
Low-Density Foam	2.25 ± 0.45	1.46 ± 0.39	1.03 ± 0.17
High-Density Foam	2.01 ± 0.37	1.52 ± 0.21	1.06 ± 0.16

**Table 2:** Average compressive properties of PET foams during first cold compression.

1° Compression	Low-Density Foam		High-Density Foam	
Direction	E_c_ (MPa)	σ_S_ (MPa)	E_c_ (MPa)	σ_S_ (MPa)
1	13.66 ± 0.92	0.71 ± 0.01	55.26 ± 4.9	2.14 ± 0.02
2	6.03 ± 0.19	0.41 ± 0.01	18.84 ± 2.1	1.08 ± 0
3	6.78 ± 1.19	0.47 ± 0.01	27.36 ± 1.43	1.05 ± 0.01

**Table 3:** Shape fixity and shape recovery after the first thermo-mechanical cycle for all foams and directions.

Direction	Low-Density Foam		High-Density Foam	
	Shape Fixity (%)	Shape Recovery (%)	Shape Fixity (%)	Shape Recovery (%)
1	58.04 ± 0.68	97.65 ± 1.18	63.77 ± 1.73	92.75 ± 0.71
2	52.91 ± 5.60	99.22 ± 1.34	59.72 ± 2.68	97.35 ± 0.80
3	53.33 ± 0.68	100 ± 0	51.80 ± 0.89	98.46 ± 0.51
